# Role of Direct Repeat and Stem-Loop Motifs in mtDNA Deletions: Cause or Coincidence?

**DOI:** 10.1371/journal.pone.0035271

**Published:** 2012-04-18

**Authors:** Lakshmi Narayanan Lakshmanan, Jan Gruber, Barry Halliwell, Rudiyanto Gunawan

**Affiliations:** 1 Institute for Chemical and Bioengineering, ETH Zurich, Zurich, Switzerland; 2 Department of Chemical and Biomolecular Engineering, National University of Singapore, Singapore, Singapore; 3 Department of Biochemistry, Neurobiology and Ageing Program, Centre for Life Sciences (CeLS), National University of Singapore, Singapore, Singapore; University of Texas Health Science Center at San Antonio, United States of America

## Abstract

Deletion mutations within mitochondrial DNA (mtDNA) have been implicated in degenerative and aging related conditions, such as sarcopenia and neuro-degeneration. While the precise molecular mechanism of deletion formation in mtDNA is still not completely understood, genome motifs such as direct repeat (DR) and stem-loop (SL) have been observed in the neighborhood of deletion breakpoints and thus have been postulated to take part in mutagenesis. In this study, we have analyzed the mitochondrial genomes from four different mammals: human, rhesus monkey, mouse and rat, and compared them to randomly generated sequences to further elucidate the role of direct repeat and stem-loop motifs in aging associated mtDNA deletions. Our analysis revealed that in the four species, DR and SL structures are abundant and that their distributions in mtDNA are not statistically different from randomized sequences. However, the average distance between the reported age associated mtDNA breakpoints and their respective nearest DR motifs is significantly shorter than what is expected of random chance in human (p<10^−4^) and rhesus monkey (p = 0.0034), but not in mouse (p = 0.0719) and rat (p = 0.0437), indicating the existence of species specific difference in the relationship between DR motifs and deletion breakpoints. In addition, the frequencies of large DRs (>10 bp) tend to decrease with increasing lifespan among the four mammals studied here, further suggesting an evolutionary selection against stable mtDNA misalignments associated with long DRs in long-living animals. In contrast to the results on DR, the probability of finding SL motifs near a deletion breakpoint does not differ from random in any of the four mtDNA sequences considered. Taken together, the findings in this study give support for the importance of stable mtDNA misalignments, aided by long DRs, as a major mechanism of deletion formation in long-living, but not in short-living mammals.

## Introduction

Mitochondria are intracellular organelles that carry multiple copies of circular double stranded DNA. Mitochondrial DNA (mtDNA) encodes multiple proteins, which are essential for ATP synthesis by the electron transport chain in the inner mitochondrial membrane. Deletion mutations have been frequently reported in mtDNA isolated from aged tissues in human and animal models [1]–[4]. High intracellular levels of mtDNA molecules with such deletions can result in respiratory chain dysfunction and ATP deficiency, leading to pathologies such as muscle fiber atrophy in sarcopenia, and have been proposed as a major factor for the decline in physiological functions in aged tissues [5], [6]. Thus, understanding how such deletions occur is essential to frame intervention strategies against pathogenic consequences of aging-associated mitochondrial dysfunction.

The precise molecular mechanism of deletion formation is not completely understood. In this regard, sequence motifs including direct repeats (DR) and stem-loops (SL) have been suggested to be involved in abnormal DNA rearrangements [7], [8]. Earlier studies have shown that a majority of deletion mutations in human mtDNA are either exactly or proximately flanked by DRs [9]–[11]. These observations have been interpreted as evidence to support the role of DRs in stabilizing mtDNA misalignments during mtDNA replication or repair that can lead to deletion formation [12]. In addition to DR motifs, SL motifs have also been reported near the deletion breakpoints in human and rat mtDNA [13], [14], but their involvement in the deletion formation is not well understood. However, the statistical significance of such observations has not been previously evaluated and considering that direct repeat motifs can be readily found in human mtDNA [9], the close proximity of deletion breakpoints to these sequence motifs can occur by random chance. Moreover, some of the observed breakpoints are not near any sequence motifs at all. Whether these motifs (DRs and SLs) are actually involved in the deletion process and whether there is any species-specific differences in the role of such motifs, is a question that demands a more careful evaluation.

In this study we have analyzed the mitochondrial genomes from human (*Homo sapiens*), rhesus monkey (*Macaca mulatta*), mouse (*Mus musculus*) and rat (*Rattus norvegicus*) for DR and SL motifs and compared them with randomly generated DNA sequences to investigate the existence of species specific or evolutionarily conserved pattern for these sequence motifs in mtDNA. Subsequently, for each species, using a set of reported age-associated mtDNA breakpoints, we explored the relationship between sequence motifs and breakpoint distributions to elucidate the role of these motifs in deletion mutagenesis. The probability of finding these sequence motifs near breakpoints purely by chance was also evaluated in each species by comparing the distribution of experimentally reported deletion breakpoints with a large set of randomly reshuffled breakpoints. During reshuffling, breakpoints were randomly placed while preserving the same total number and deleted lengths as those from the reported deletions. Using these data, we also addressed any likely differences in the mtDNA deletion mutagenesis between short-living and long-living species.

## Results

### Abundance, Distribution and Stability of DR Motifs in Mitochondrial Genomes

**Figure 1:**
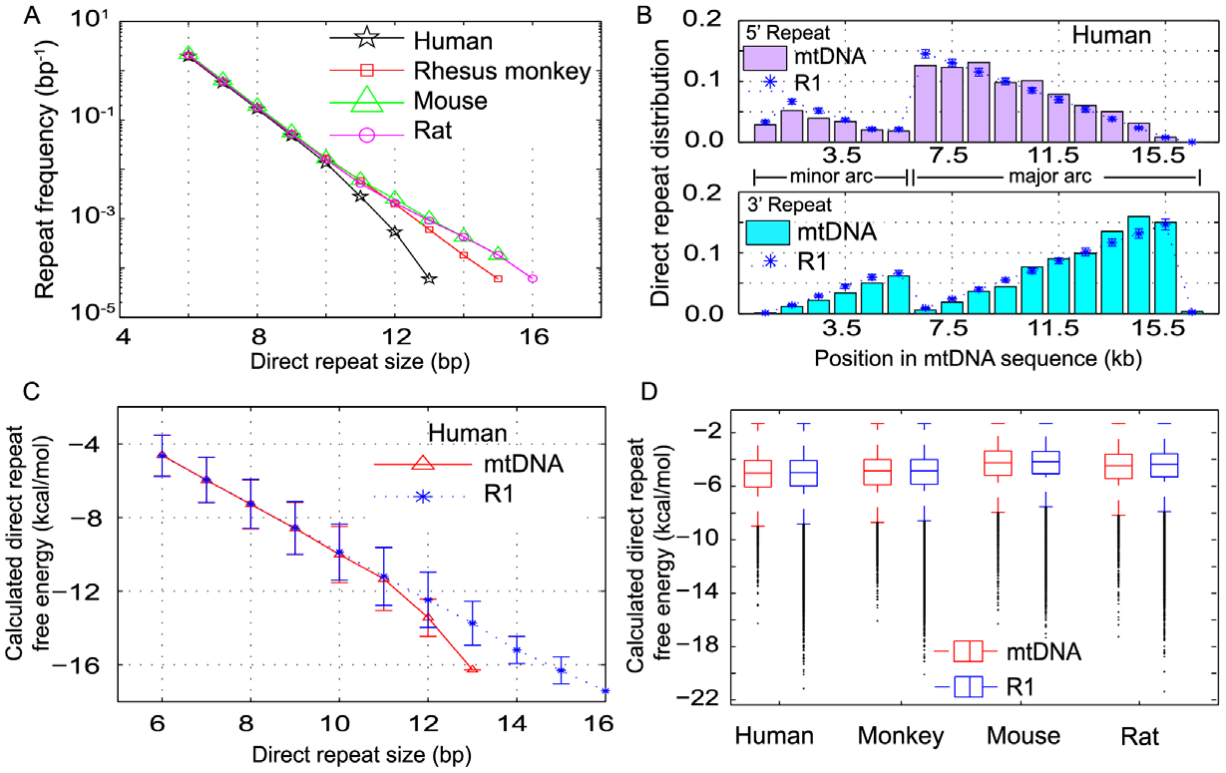
Analysis of direct repeat (DR) motifs in mitochondrial genomes. Abundance, distribution and free energy of DRs in mtDNA and random DNA sequences (*n* = 100) with the same base composition (R1) as corresponding mtDNA. (A) Frequency of DR pairs (≥6 bp) in the mtDNA of human, rhesus monkey, mouse and rat. The DR frequency is normalized with respect to the mtDNA length in each species. (B) The distribution of left and right DR sequences in the minor and major arcs of human mtDNA and the mean distribution of DRs in R1 random sequences. (C) DR sizes and DR free energies in human mtDNA and the corresponding R1 random sequences. The lower the free energy of a DR, i.e. the more negative, the more stable is the DNA duplex formed. (D) Distribution of free energies of DNA duplex formed by DRs (≥6 bp) in native mtDNA and random sequences (R1) of human, rhesus monkey, mouse and rat.

The analysis of all four mtDNA sequences revealed an abundance of direct repeats of various sizes in the mitochondrial genome, where roughly 80% of the DRs resided in the major arc. As expected from simple probability, the DR frequencies generally decreased exponentially with DR size (Figure 1A). The maximum size of DR observed was 13 bp in human, 15 bp in rhesus monkey and mouse, and 16 bp in rat mtDNA. In comparison to random sequences of the same length and with the same base composition (R1 sequences, *n* = 100), the mtDNA of human, rhesus monkey, mouse and rat possess relatively higher DR counts for every DR size ≥6 bp (z-test, p≤0.0061) (Figure S1), except for large DRs of size ≥11 bp and ≥14 bp in the case of human and monkey, respectively (p≥0.073). Random DNA sequences with equal proportions of each of the four nucleotides (R2 sequences) had lower DR frequencies than either native mtDNA for each DR size ≥6 bp (z-test, p≤0.0057) or R1 sequences (two sample t-test, p≤0.0015). The only exception was the 13 bp DR (p = 0.4467) in native human mtDNA. By comparing the DR frequency distribution across species (Figure 1A), we noted a trend toward fewer large DRs (>11 bp) with the longer lifespan of the organisms, which is consistent with an earlier study showing that the frequencies of long DRs (>10 bp) are inversely correlated with the lifespan of the organisms [15]. In particular, the frequencies of the longest DRs in human were lower than those in mouse or rat, beyond what is expected from frequency differences between random R1 sequences from each respective organism (p<0.01; see Figure S2, S3, S4).

The probability of finding a left (5′) direct repeat had a peak at the beginning of the major arc sequences, and this is expected since the chance of finding an identical repeat sequence depends on the length of the remaining sequence downstream of this putative repeat. Correspondingly, the probability of finding a right (3′) direct repeat was high near the D-loop region. There was no obvious difference between the distribution of DRs in native mtDNA and random R1 sequences in any of the four species, indicating a random pattern of distribution of DR motifs in the mitochondrial genomes (Figure 1B for human; Figure S5 for rhesus monkey, mouse and rat).

Misalignments between DRs during mtDNA replication or repair have been implicated in mtDNA deletions [12]. To investigate the stability of such putative misalignments, we calculated the free energies of DNA duplexes formed by each DR [16]. The Gibb's free energy of hybridization is sequence specific and is an inherent property of any DNA sequence. In the four mtDNA, the free energies for all DRs of size ≥6 bp ranged between −4 to −88 kJ/mol (or −1 to −21 kcal/mol). Generally, there exists a linear relationship between the size of DR and the free energy, i.e. the longer the DR, the lower is the free energy and the more stable is the DNA duplex (Figure 1C for human; Figure S6 for rhesus monkey, mouse and rat). The distribution of the free energies in mtDNA was skewed due to the existence of a large number of short and less stable DRs. Again, the distribution and median free energies in native mtDNA differed little from those in random R1 sequences in any four species (Figure 1D). The 13 bp DR in human mtDNA had a lower free energy than the average of random R1 sequences but the deviation was not statistically significant (z-test, p = 0.083).

### Stem-loop Motifs in Mitochondrial Genomes

It has been suggested that the major arc of the heavy-strand (H-strand) of mtDNA may temporarily exist in a single stranded form during mtDNA replication (Strand Asynchronous model), and that in this state, DNA misalignment can occur, possibly leading to deletion mutation [17],[18]. Stem-loop structures may stabilize the single-stranded H-strand and thus increase the chance for such misalignment. On the other hand, these motifs could also prevent DNA hybridization, thereby protecting the mtDNA from strand misalignment. Thus, the role of SLs in mtDNA deletion mutagenesis is ambiguous.

**Figure 2:**
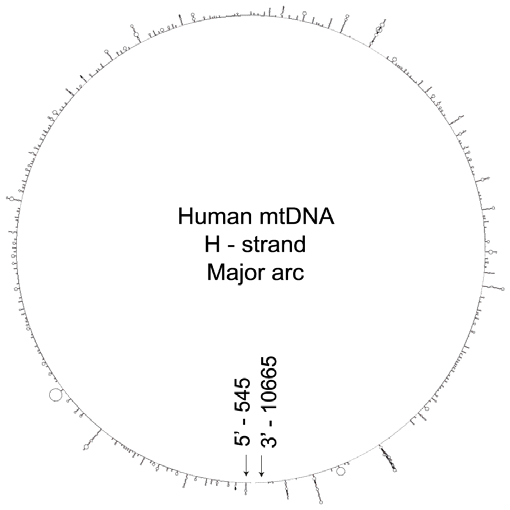
Stem-loop (SL) motifs in human mitochondrial genome. Abundance and distribution of predicted SL motifs in single-stranded human mtDNA heavy strand major arc. The positions 545 to 10665 correspond to the H-strand sequence from the end of D-loop till the beginning of L-strand origin of replication respectively. The minimum free energy folded structure is depicted in circular form.

Similar to the DRs, the analysis of all four major arc H-strands revealed a large number of SL structures (Figure 2 for human; Figures S7, S8, S9for rhesus monkey, mouse and rat), where more than one third of the nucleotides in the major arcs could form stems (Table 1). The comparison between the analysis of SL motifs in the native mtDNA and R1 (*n* = 5) showed that SLs were equally abundant in native and random sequences in human, mouse and rat (two sided t-test, p≥0.0317), but not in rhesus monkey (p = 0.0001). In addition, there was no specific pattern of distribution that could be discerned for the mtDNA SL motifs from the four species. Thus, these findings suggest that SL motifs may not be an important evolutionary factor in mtDNA.

**Table 1:** Abundance of stem-loop motifs in mtDNA and random DNA sequences.

Species	Percentage of major arc forming stems		
	mtDNA	Random DNA	Two sided t-test
		(R1; *n* = 5)	*p* – value
Human	37.05	38.38±0.91	0.0317
Rhesus Monkey	34.77	38.04±0.51	0.0001
Mouse	37.97	39.15±1.27	0.1052
Rat	39.88	39.15±0.70	0.0821

### Age associated mtDNA Deletion Breakpoint Spectra in Humans and Animal Models

Because of the abundance of DR and SL motifs in mtDNA and the similarity between the distribution of DRs and SLs in native and in random sequences, one might argue that the proximity of mtDNA deletion breakpoints to any of these motifs could simply be random occurrences. To establish a causal role, if any, of these motifs in deletion formation, we first compared the distribution of sequence motifs with the distribution of unique, experimentally determined deletion breakpoints.

**Figure 3:**
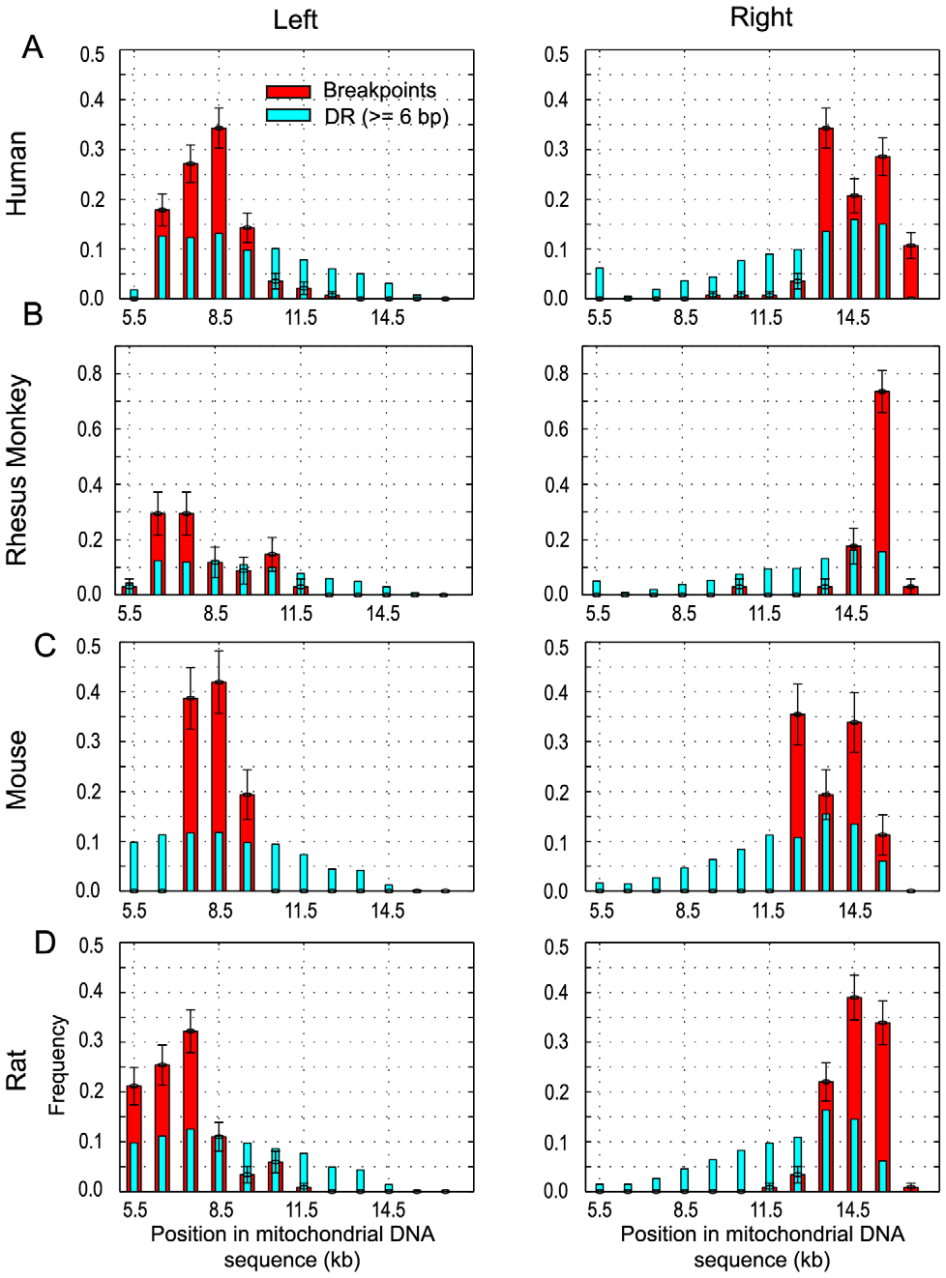
Deletion breakpoints and direct repeats. Distributions of left and right breakpoints of aging-associated mtDNA deletions and of left and right DR motifs from (A) human, (B) rhesus monkey, (C) mouse and (D) rat. Standard deviation (error bars) for the frequency of breakpoints in each bin is calculated based on a binomial distribution.

We compiled 354 distinct, age-associated mtDNA deletions that had previously been observed experimentally in human (*n* = 140), rhesus monkey (*n* = 34), mouse (*n* = 62) and rat (*n* = 118), from the literature (see Document S1). Data on the distribution of deletion breakpoints in human mtDNA had been reported earlier [9] and our results were in good agreement with this result. In general, the left breakpoints of the reported deletions crowded near the beginning of the major arc and the right breakpoints were localized near the D-loop region (see Figure 3). Comparison of the breakpoint positions with the distribution of DRs in the four mammals revealed that the peaks of the breakpoint distributions colocalized with the mtDNA regions of high DR frequency. However, it is important to note that such colocalization alone is not sufficient to prove the involvement of DR in the deletion formation, as the same is true even if we use randomly generated instead of the actual mtDNA sequences (see Figure 1B). The reason for this superficially surprising result is that the probability of finding complementary repeats of a given nucleotide sequence depends on the maximum length of the up- and downstream sequences (i.e. the search space) and thus, the maxima of such probability expectedly lie at both ends of any sequence.

**Figure 4:**
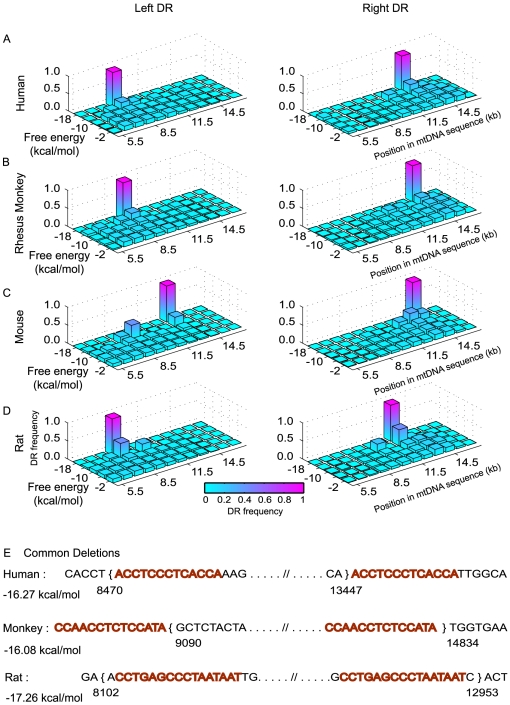
Free energy and position wise distribution of the DRs in mtDNA major arc. Resolution of DR distribution based on DR free energy and position in mtDNA sequence of (A) human, (B) rhesus monkey, (C) mouse and (D) rat. The x- and y- axis values denote the midpoint of each corresponding bin, i.e. a bin centered at 5.5 kb denotes a range from 5 to 6 kb and similarly, a bin centered at −2 kcal/mol has a range between 0 to −4 kcal/mol. (E) The most stable DR motifs in mtDNA major arc are associated with reported common deletions. Left and right breakpoint positions (denoted by open and close braces respectively) of common deletion [18], [27], [28], the flanking DR sequence (highlighted in red) and the calculated DR free energy value in human, rhesus monkey and rat mtDNA.

As higher stability of DNA duplex misalignments had been implied to correlate with increased occurrences of deletions in human [19], we then organized the DR distribution in mtDNA based on increasing free energy, as shown in Figure 4. Using this approach, we observed that in human mtDNA, the regions with high frequency of breakpoints (left breakpoint frequency peaking at ∼8.5 kbp and right breakpoint frequency peaking at ∼13.5 kbp and ∼15.5 kbp) coincided with segments of the native mtDNA with higher density of low free energy (more stable) DRs (Figure 4A), but not with the stable DRs in random R1 sequences (Figure S10). Interestingly, the most stable DR motifs in the mtDNA major arc were associated with the reported “common deletion", i.e. the most frequently reported deletions, in human, rhesus monkey and rat coincide with the most stable repeats in the respective mtDNA sequences (Figure 4E). Thus, despite the abundance of DR, the association between common deletions and the most stable repeats supports an important role of mtDNA misalignments due to DR in the formation of deletions. On the contrary, the stability of complementary mtDNA sequences has been previously shown to correlate with lifespan, where short living species have higher average free energy values (less stable DNA hybridization) than long living species [20]. In this case, the lesser stable mtDNA are hypothesized to have increased probability of forming random (small) DNA bubbles, leading to enhanced (point) mutation rates.

To further establish the role of DRs in the formation of mtDNA deletions, for each reported deletion, we computed the average distance between the breakpoints and the nearest DR motif (see methods). In addition, we also generated a set of “reshuffled" breakpoints by randomly placing deletions within the major arc, while preserving the deletion length distribution. The proximity of these randomly placed breakpoints to any DRs clearly arises by chance. By comparing breakpoint-to-nearest-DR proximity values from the reported deletions with those from the reshuffled deletions, we are able to show that the experimentally observed mtDNA deletions are statistically significantly closer to a DR than what is expected by random chance in human (p<10^−4^) and rhesus monkey (p = 0.0034), but not in mouse (p = 0.0719) and rat (p = 0.0437). The same general observations held true when such comparisons were done using subsets of mtDNA deletions data from individual papers and from specific tissues, indicating little or no methodological and tissue bias in the above finding (see Document S2).

In contrast to the DRs, there was no discernable relationship between the SL structures and reported deletion breakpoints. As shown in Table 1, the double stranded stem regions were formed by 30–40% of nucleotides in the major arc H-strand, while similar fractions of the reported breakpoints were found within these structures (Table 2). The fraction of randomly reshuffled breakpoints found in stem regions was also similar to that of reported breakpoints in the four mammals (p≥0.0160). Despite a previous report suggesting an involvement of SL in deletion mutagenesis [14], we found no strong evidence for the role of SLs in either promoting or preventing deletion mutagenesis in these organisms (rhesus monkey, mouse and rat). In this case, the occasional presence of such motif near the breakpoints is indistinguishable from what would be expected by random chance, a fact that only became apparent when the reported breakpoints are compared with the reshuffled breakpoints, illustrating the value of using randomly generated datasets as reference.

**Table 2:** Role of sequence motifs in mtDNA deletions.

Species	Percentage breakpoints within stem regions			Average breakpoint – nearest DR distance		
	Reported	Reshuffled	Two sided z-test	Reported	Reshuffled	z-test
		(*n* = 100)	*p* - value		(*n* = 100)	*p-value*
Human	29.28	37.4±3.37	0.0160	10.39	17.37±0.80	<10^−4^
R. Monkey	33.82	35.44±5.49	0.7679	12.51	17.32±1.77	0.0034
Mouse	41.93	38.03±4.42	0.3776	15.59	17.24±1.12	0.0719
Rat	39.40	40.63±2.80	0.6605	16.53	18.20±0.97	0.0437

## Discussion

Accumulation of mitochondrial DNA deletions is implicated in many degenerative and age-related diseases in humans, including Parkinson's disease, Kearn-Sayre syndrome, and sarcopenia [1], [21]–[23]. Such mutations have been attributed to the misalignment of mtDNA during replication or repair, a hypothesis that was supported by frequent observations of genome motifs, particularly direct repeat, flanking or near the deletion breakpoints in human and other species [11], [24], [25]. However, as there exist many DRs in native mtDNA, the proximity of any breakpoints to a DR could potentially arise by random chance. In addition to DR, in this study we have also analyzed the frequency and distribution stem loop structures, another genome motif that has been previously implicated in mtDNA deletion formation [9], [14]. We compare the results from the analysis of mtDNA of human, monkey, mouse and rat, to establish the validity of different mutation etiology hypothesis and to investigate any interspecies differences.

There are three key observations from the comparative analysis of DR distributions. The first is that DRs can be readily found in native mtDNA and random DNA sequences, more so in the former than in the latter. It should be noted that the higher frequency for DRs (size ≤10 bp) in native mtDNA over the random sequences could be due to the fact that native sequences encode proteins and thus have additional constraints related to codon usage and protein motifs. Second, left and right DRs are concentrated at the beginning and the end of the major arc in both native and random sequences, respectively, and the positions of these hotspots arise, as expected, from simple probability. While the distributions of the reported breakpoints also share the same features, this similarity does not necessarily imply causality. Finally, there exist trends towards lower frequencies of longer and thus more stable DRs and towards smaller maximum DR size with longer-living mammals, i.e. human and monkey. As these trends are absent among the random (R1) mtDNA sequences, this finding suggests a possible evolutionary selection pressure against long, stable DRs (>10 bp) in long-living (human and monkey), but not in short-living mammals of the four studied here (mouse and rat).

The crucial support for the causal role of DRs in the formation of mtDNA deletions in human and monkey came from comparing the average distances to the nearest DR from the reported and randomly reshuffled breakpoints. In the case of mtDNA deletions from human and rhesus monkey, DRs are found at much shorter distances from the breakpoints than expected by random chance. Taken together, the lower proximate distance to DRs and the lower frequencies of long (stable) DRs implicate this genome motif in deletion mutagenesis in the longer-living mammals among the four. This finding is also consistent with an earlier report showing that deletion breakpoints are more likely to occur within highly stable mismatched duplexes (100 bp) in aged human tissues [19]. Such stable mismatched duplexes could be formed by multiple consecutive DR motifs. In addition, mtDNA regions flanking deletion breakpoints of human patients suffering from mitochondrial diseases have been found to have an increased sequence homology [26]. In contrast to DR, we found no evidence of SL involvement in mtDNA deletion formation in any of the four mammals.

Our analysis of mouse and rat mtDNA, however, revealed a lack of significant evidence for a major involvement of DR in the deletion mutagenesis in these short-living mammals. The difference between short- and long-living mammals here alludes to another mode (or modes) of mutagenesis, which may be dominant during the developmental stages of organisms and is likely driven by errors during mtDNA replications in development. Meanwhile, mtDNA misalignments involving DRs could conceivably cause deletions later in life. In support of this hypothesis, mitochondrial DNA deletions from young and old humans with mitochondrial diseases were previously shown to have different characteristics, where sequence homology was higher in the neighborhood of deletion breakpoints from the old than that from the young patients [26]. Hence, studies on human aging on the basis of mtDNA deletions using short-living model organisms should appreciate the potential difference in the etiology of mutagenesis.

In view of the possibility of two distinct origins of mtDNA deletions: developmental and somatic, the latter should predominantly affect long-living organisms. Consequently, deletions induced by DNA misalignments may be evolutionarily selected against in long-living organisms, as there is a higher chance for such deletions to (clonally) accumulate in these animals than in short-living ones. As mentioned earlier, our analysis revealed an interspecies trend toward lower frequencies of larger and more stable DRs with longer lifespan, which is in agreement with an earlier report [15] and is also consistent with the hypothesized selection pressure against stable mtDNA misalignments. However, as the analysis here was done only for four species from the mammals, further studies using more species will be needed to establish the generality of the findings presented here. Naturally, there may also exist a selection against deletions of other origins, but this could not be checked from the motif analysis in our study.

## Materials and Methods

### Mitochondrial DNA and random DNA sequences

The mitochondrial DNA (mtDNA) sequences used in this study were obtained from the National Centre for Biotechnology Information (NCBI) database. The reference numbers and other information regarding the mitochondrial genomes of human and the three other model organisms are given in Table 3. Rhesus monkey, mouse and rat were chosen on the basis that these three species are among the frequently used mammalian model organisms in the studies on mtDNA deletions and aging, and a sufficiently large number of aging associated deletions have been reported for them. Random R1 sequences (*n* = 100) were generated for each native mtDNA with the same length and nucleotide base composition as the respective mtDNA, using ***randseq*** subroutine available from the Bioinformatics toolbox in MATLAB. In the same manner, random R2 sequences (*n* = 100) were generated with the same length as each native mtDNA, but with equal proportions of the four nucleotides (i.e. 25% each).

**Table 3:** Mitochondrial genome sequence information.

Animal	GenBank Reference	L-strand regions used for DR scan		H-strand regions used for SL motif prediction (5′ – 3′)
		Minor arc	Major arc	
*Homo sapiens*	NC_012920	577 – 5890	5905 - 16023	545 – 10665
*Macaca mulatta*	NC_005943	536 – 5680	5714 – 16014	549 – 10851
*Mus musculus*	NC_005089	1 – 5159	5192 – 15422	876 – 11108
*Rattus norvegicus*	X14848	1 – 5139	5171 - 15403	896 – 11130

### Analysis of direct repeats in mitochondrial genomes

The minor and major arc regions of mtDNA light strand (L-strand) of each species were separately scanned for the presence of DRs of length longer than 6 bp. Smaller DRs (≤5 bp) were not considered in the study as (1) they were too large in number, resulting in overcrowding of distribution plots (affecting the clarity of the results), and importantly (2) a large majority of these DRs have a positive DNA duplex free energy (i.e. they do not readily hybridize). For each identified DR pair, the size and positions of the left (5′) and right (3′) repeats were recorded. Subsequently, the histograms of the left and right DRs were generated using 17 bins of size 1000 bp, i.e. 1–1000, 1001–2000, and so on. This binning scheme was used throughout the study. Direct repeat pairs which encompass L-strand origin or D-loop were not included in the study. The analysis of random sequences was also separated into major and minor arc (for direct repeat scanning) using same L-origin and D-loop positions as the respective native mtDNA. The list of DRs (≥6 bp) included overlapping repeats, i.e. shorter repeats within longer repeats and partially overlapping repeats. However, accounting only non-overlapping DR gave the same conclusion with DRs distributed throughout the minor and major arcs of mtDNA in all four species (results not shown).

The ***hybrid-min*** program from the UNAFold package was used with the default parameter settings in the calculations of the free energy of hybridization between the 5′ – 3′repeat sequences and its reverse complement [16].

### Analysis of stem loop motifs in mitochondrial genomes

The DNA folding algorithm ***hybrid-ss-min*** from UNAFold package [16] was used with the default parameter settings to predict the minimum energy folded structure of the entire single stranded heavy strand from the end of D-loop till the beginning of L-strand origin of replication in each mtDNA. In this case, the folding calculation was done separately for every consecutive 1000 bp regions of the H-strand. However, there was no difference in the predicted stem loop structures when compared to the folding calculation using the entire H-strand region from the end of D-loop till the beginning of L-strand origin of replication. The percentage of nucleotides involved in double-stranded stems and the number of reported breakpoints which are found in the stem regions were counted using this predicted minimum free energy structure. For R1 sequences, the H-strands were derived from the L-strand random sequences and then the same folding calculations as above were performed.

### Analysis of deletion breakpoints

#### Aging associated deletion breakpoints in mitochondrial genomes

Breakpoint positions of aging associated mtDNA deletions were collected from the literature (see Document S1). In this study, we have considered only aging associated mtDNA deletions (deletions from myopathy patients and other clinical conditions were not included in this study) that occurred within the major arc, as the numbers of deletions in the minor arcs were much smaller. Identical deletions had been reported in different research articles, e.g. the common deletion, and here such deletions were counted only once. In addition, two or more deletions with left and right breakpoints within 5 bp from each other, for example deletion1: 8469–13447 and deletion2: 8468–13445, were again counted once. In this manner, a set of non-redundant deletions were compiled for each species. The distributions of left and right breakpoints were created by histograms with the same binning scheme as that for the DRs. The error-bars represented the standard deviations, which were calculated by the following formula (assuming a binomial probability distribution for each bin): 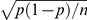 where *n* is the total number of (left or right) breakpoints and *p* is the frequency of breakpoints in an individual bin.

#### Calculation of average distance between breakpoints and the nearest DR

For each deletion, the nearest DR pair is one with the minimum total distance to the left and right breakpoints. In the analysis, we have used the average distance, i.e. the total distance divided by two, as the DR proximity value.

#### Reshuffled Deletions

The reshuffling of mtDNA deletions was done by randomly placing the left breakpoint of non-redundant deletions with equal probability in the feasible region of the major arc (such that the deletion still stays within the major arc). In total, 100 sets of randomly reshuffled deletions were generated.

## Supporting Information

Figure S1 **Frequency of direct repeat pairs (≥6 bp) in mtDNA.** Frequency of direct repeat pairs in mtDNA and two types of random sequences (R1 and R2) in (a) human, (b) rhesus monkey, (c) mouse and (d) rat. R1 denote the random sequences with the same base composition as the corresponding mtDNA. R2 denote random sequences with equal proportion of all four bases.
(TIF)

Figure S2 **Frequency difference distribution of direct repeats (DR).** Frequency difference distribution of DR between random R1 sequences from human and from rat (100 R1 from each or 10,000 total differences) for DR sizes from 6 to 13 bp. The frequency difference between the native human and rat mtDNA (**|||**) is also shown with the p-value (2-sided, z-test) noted in each subfigure.
(TIF)

Figure S3 **Frequency difference distribution of direct repeats (DR).** Frequency difference distribution of DR between random R1 sequences from rhesus monkey and from rat (100 R1 from each or 10,000 total differences) for DR sizes from 6 to 15 bp. The frequency difference between the native rhesus monkey and rat mtDNA (**|||**) is also shown with the p-value (2-sided, z-test) noted in each subfigure.
(TIF)

Figure S4 **Frequency difference distribution of direct repeats (DR).** Frequency difference distribution of DR between random R1 sequences from mouse and from rat (100 R1 from each or 10,000 total differences) for DR sizes from 6 to 15 bp. The frequency difference between the native mouse and rat mtDNA (**|||**) is also shown with the p-value (2-sided, z-test) noted in each subfigure.
(TIF)

Figure S5 **The distributions of left and right DR sequences (≥6 bp).** The distributions of left and right DR in the mtDNA minor and major arcs and the mean distributions of R1 random sequences in (a) human, (b) rhesus monkey, (c) mouse and (d) rat.
(TIF)

Figure S6 **DR sizes and free energies.** DR sizes and free energies in native mtDNA and the corresponding R1 random sequences of (a) human, (b) rhesus monkey, (c) mouse and (d) rat.
(TIF)

Figure S7 **Stem-loop (SL) motifs in rhesus monkey mitochondrial genome.** Abundance and distribution of predicted SL motifs in single-stranded rhesus monkey mtDNA heavy strand sequence from the end of D-loop till the beginning of L-strand origin of replication respectively. The minimum free energy folded structure is depicted in circular form.
(TIF)

Figure S8 **Stem-loop (SL) motifs in mouse mitochondrial genome.** Abundance and distribution of predicted SL motifs in single-stranded mouse mtDNA heavy strand sequence from the end of D-loop till the beginning of L-strand origin of replication respectively. The minimum free energy folded structure is depicted in circular form.
(TIF)

Figure S9 **Stem-loop (SL) motifs in rat mitochondrial genome.** Abundance and distribution of predicted SL motifs in single-stranded rat mtDNA heavy strand sequence from the end of D-loop till the beginning of L-strand origin of replication respectively. The minimum free energy folded structure is depicted in circular form.
(TIF)

Figure S10 **Free energy and position-wise distribution of the DRs.** Resolution of DR distribution based on DR free energy and position in R1 random sequences (*n* = 100) of human mtDNA. The x- and y- axis values denote the midpoint of each corresponding bin, i.e. a bin centered at 5.5 kb denotes a range from 5 to 6 kb and similarly, a bin centered at −2 kcal/mol has a range between 0 to −4 kcal/mol.
(TIF)

Document S1 Breakpoints data with reference.
(PDF)

Document S2 Average breakpoint-nearest DR distances in breakpoint data from individual studies.
(PDF)
