# Supplementary figures and images for: Role of Direct Repeat and Stem-Loop Motifs in mtDNA Deletions: Cause or Coincidence?
Source: PLoS One. 2012 Apr 18;7(4):e35271. doi: 10.1371/journal.pone.0035271 (PMC3329436; doi:10.1371/journal.pone.0035271)

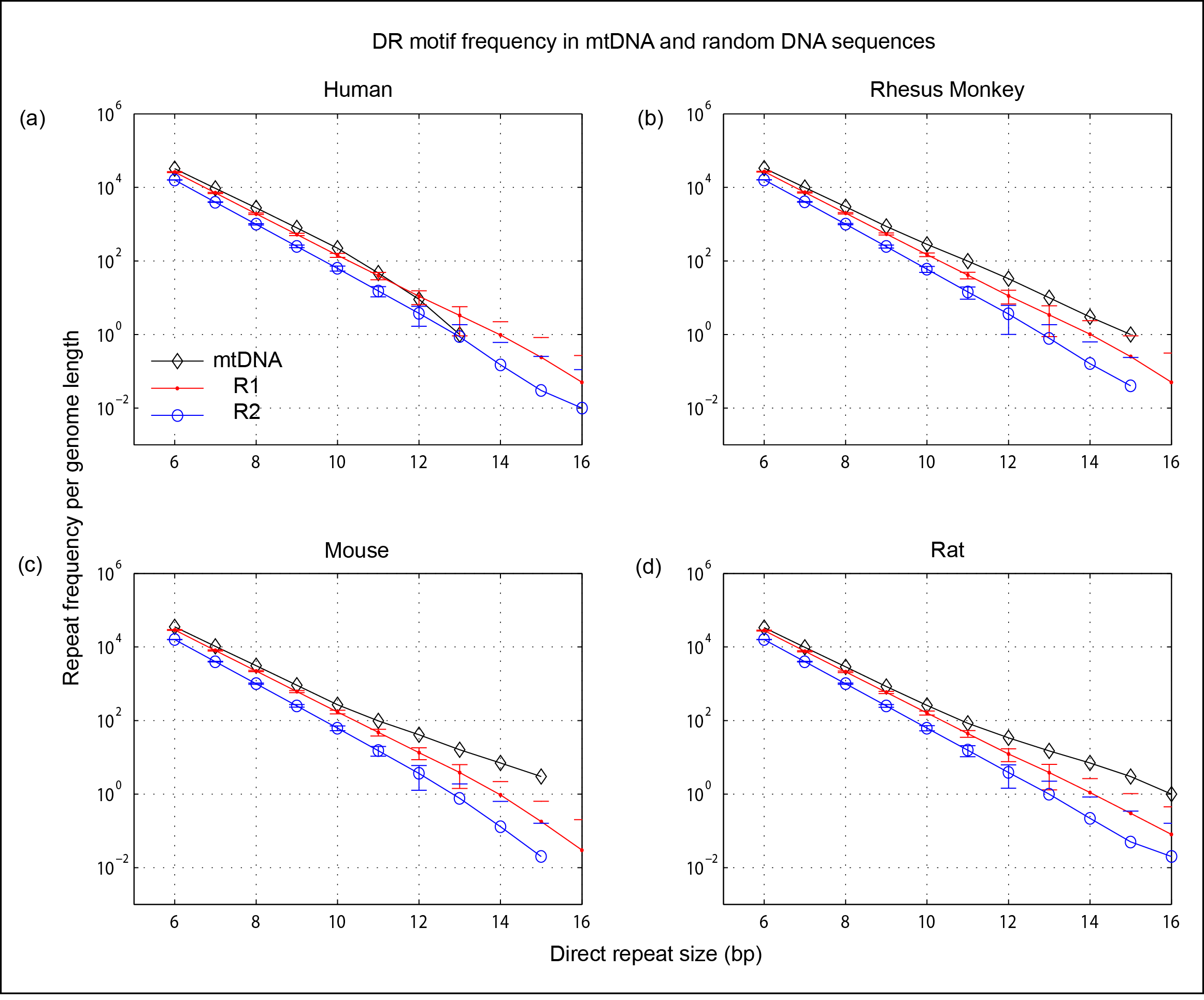

Supplement: Figure S1 — Frequency of direct repeat pairs (≥6 bp) in mtDNA. Frequency of direct repeat pairs in mtDNA and two types of random sequences (R1 and R2) in (a) human, (b) rhesus monkey, (c) mouse and (d) rat. R1 denote the random sequences with the same base composition as the corresponding mtDNA. R2 denote random sequences with equal proportion of all four bases. (TIF) [file pone.0035271.s001.tif]

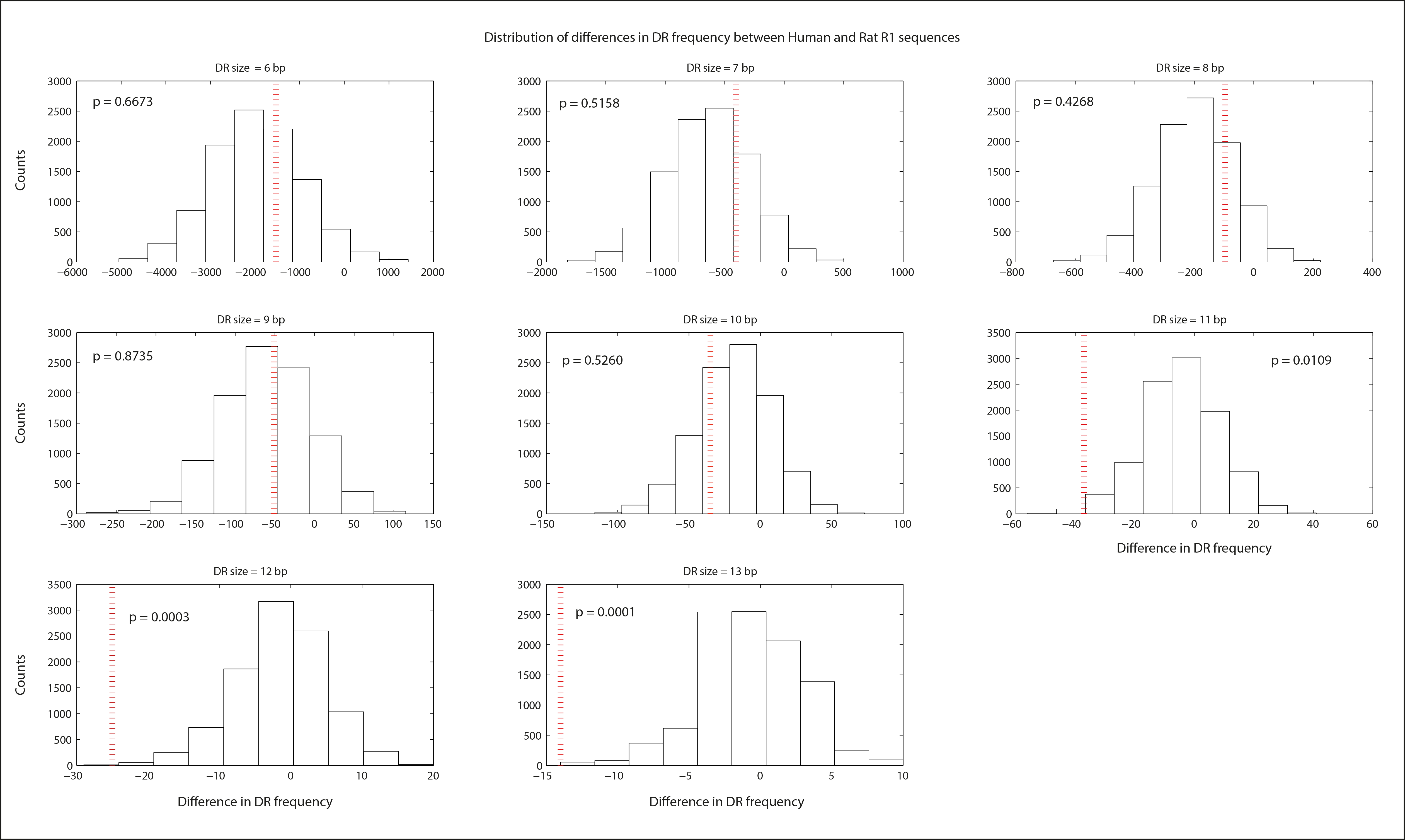

Supplement: Figure S2 — Frequency difference distribution of direct repeats (DR). Frequency difference distribution of DR between random R1 sequences from human and from rat (100 R1 from each or 10,000 total differences) for DR sizes from 6 to 13 bp. The frequency difference between the native human and rat mtDNA (|||) is also shown with the p-value (2-sided, z-test) noted in each subfigure. (TIF) [file pone.0035271.s002.tif]

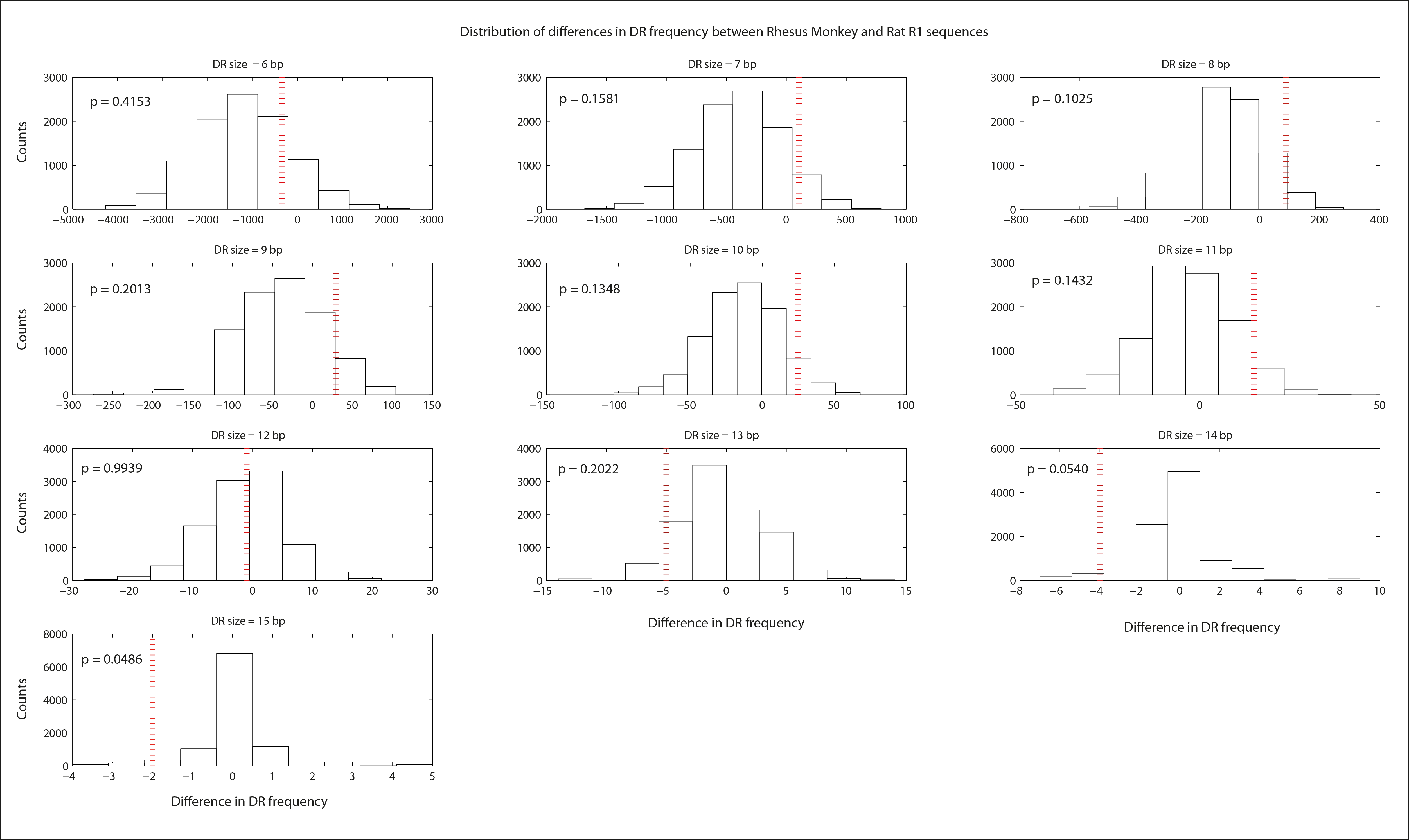

Supplement: Figure S3 — Frequency difference distribution of direct repeats (DR). Frequency difference distribution of DR between random R1 sequences from rhesus monkey and from rat (100 R1 from each or 10,000 total differences) for DR sizes from 6 to 15 bp. The frequency difference between the native rhesus monkey and rat mtDNA (|||) is also shown with the p-value (2-sided, z-test) noted in each subfigure. (TIF) [file pone.0035271.s003.tif]

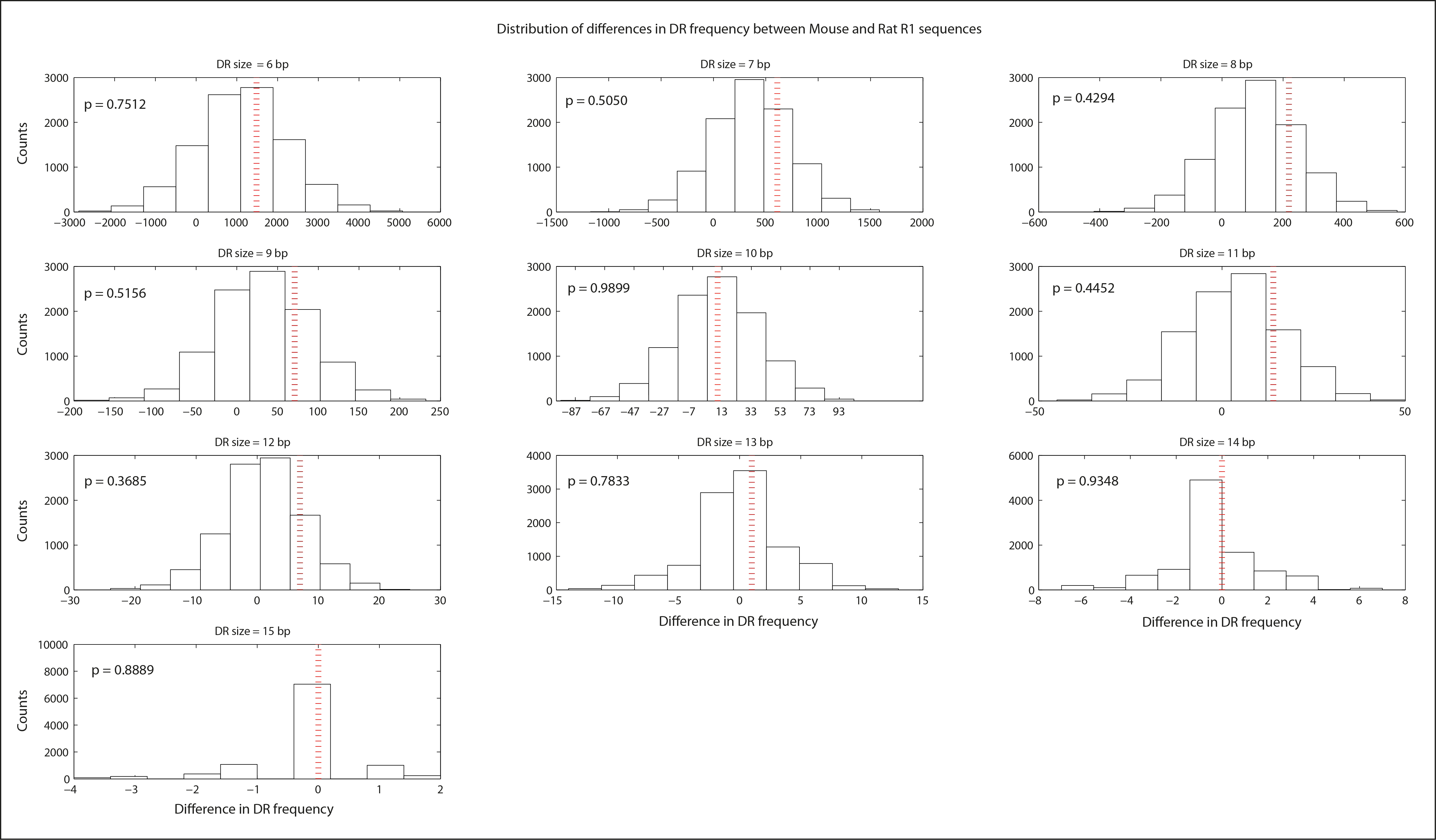

Supplement: Figure S4 — Frequency difference distribution of direct repeats (DR). Frequency difference distribution of DR between random R1 sequences from mouse and from rat (100 R1 from each or 10,000 total differences) for DR sizes from 6 to 15 bp. The frequency difference between the native mouse and rat mtDNA (|||) is also shown with the p-value (2-sided, z-test) noted in each subfigure. (TIF) [file pone.0035271.s004.tif]

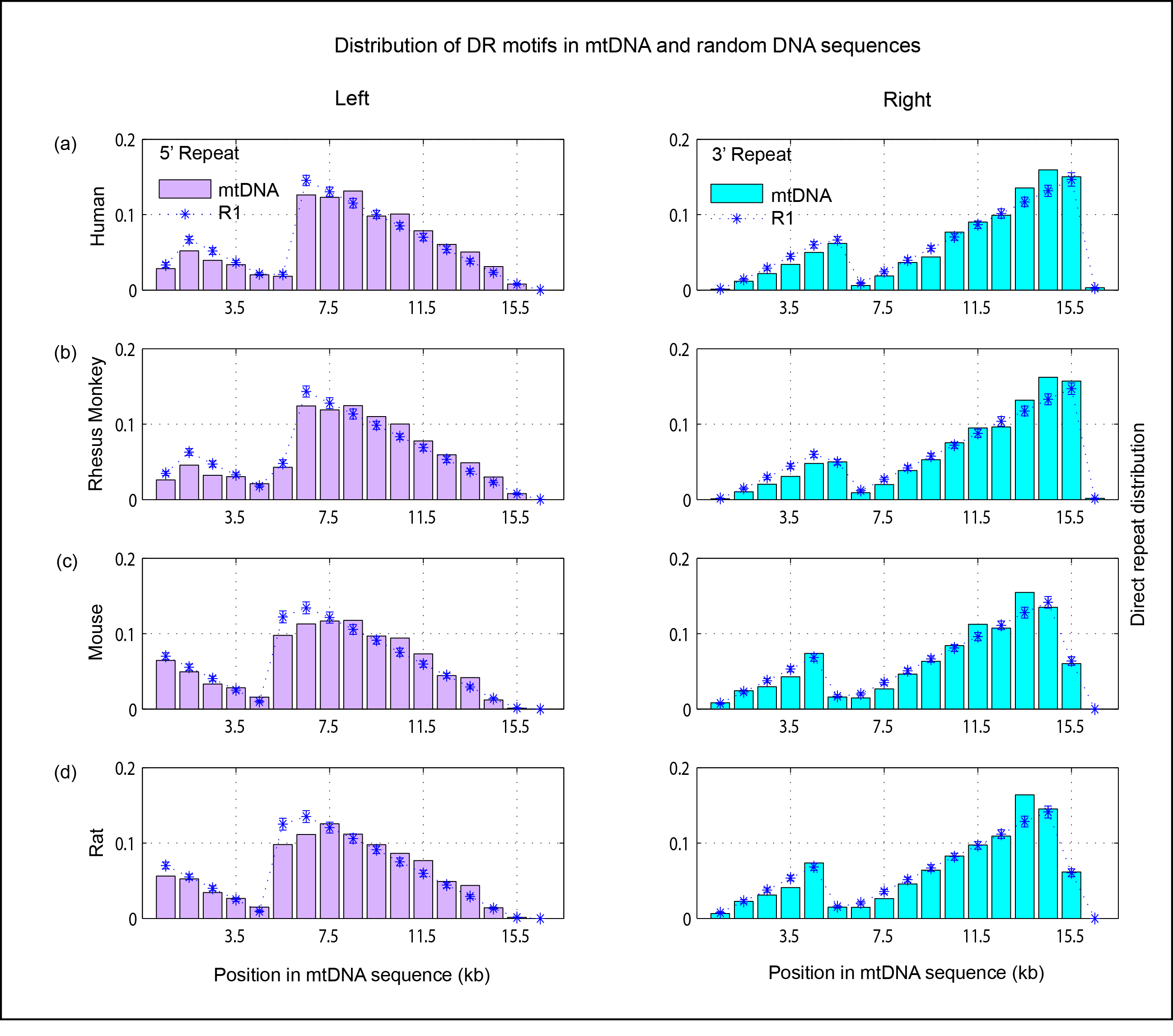

Supplement: Figure S5 — The distributions of left and right DR sequences (≥6 bp). The distributions of left and right DR in the mtDNA minor and major arcs and the mean distributions of R1 random sequences in (a) human, (b) rhesus monkey, (c) mouse and (d) rat. (TIF) [file pone.0035271.s005.tif]

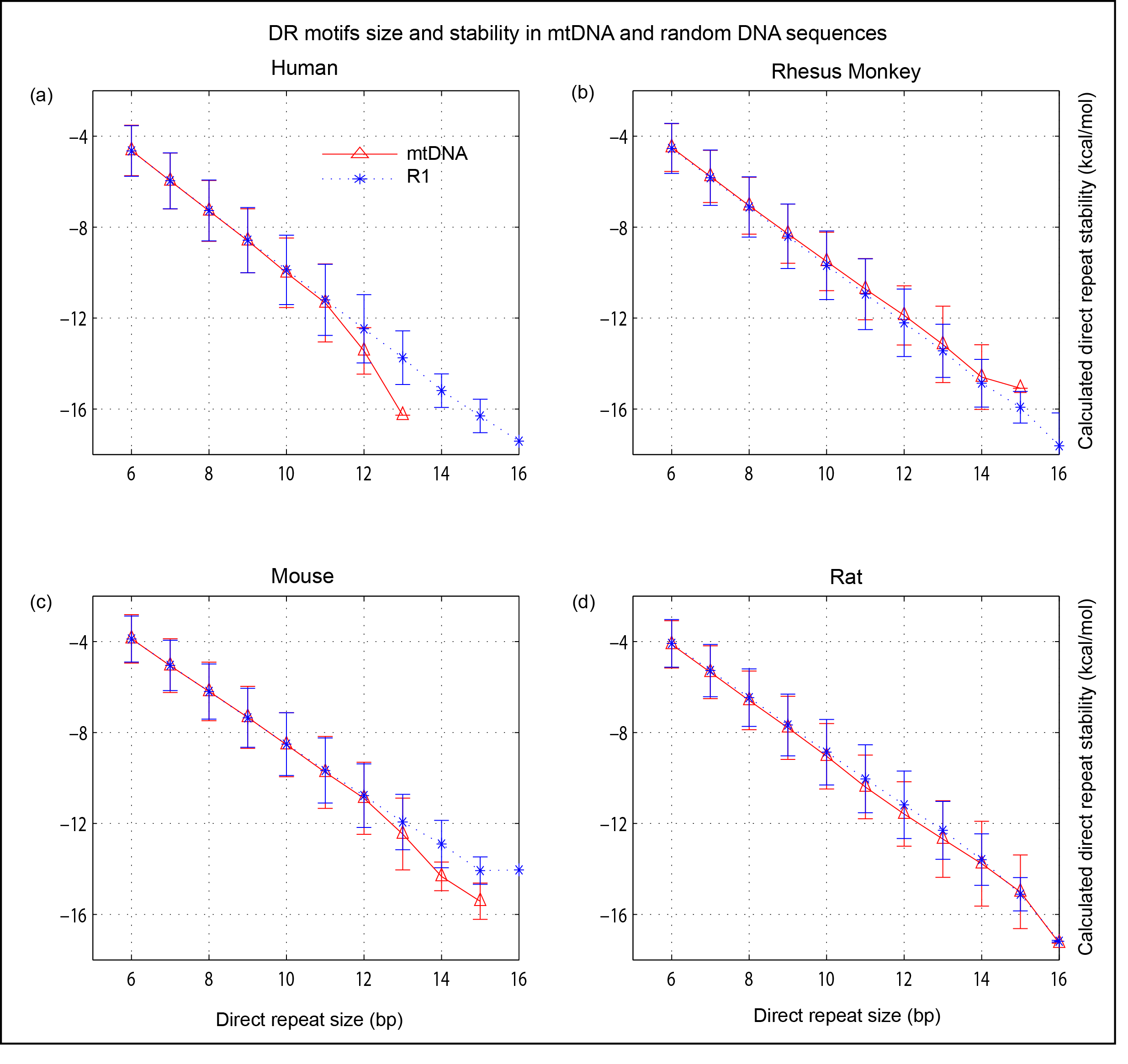

Supplement: Figure S6 — DR sizes and free energies. DR sizes and free energies in native mtDNA and the corresponding R1 random sequences of (a) human, (b) rhesus monkey, (c) mouse and (d) rat. (TIF) [file pone.0035271.s006.tif]

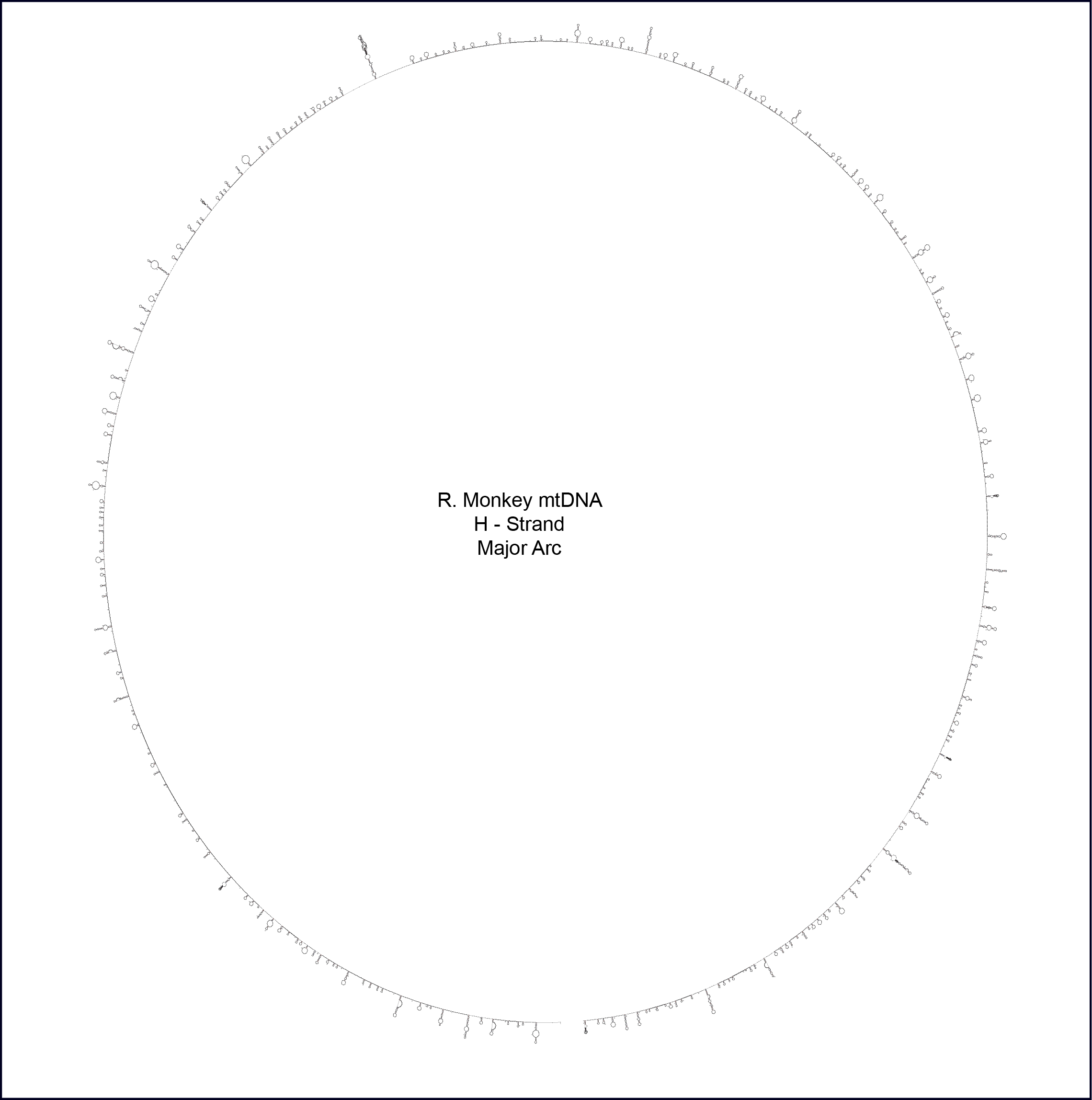

Supplement: Figure S7 — Stem-loop (SL) motifs in rhesus monkey mitochondrial genome. Abundance and distribution of predicted SL motifs in single-stranded rhesus monkey mtDNA heavy strand sequence from the end of D-loop till the beginning of L-strand origin of replication respectively. The minimum free energy folded structure is depicted in circular form. (TIF) [file pone.0035271.s007.tif]

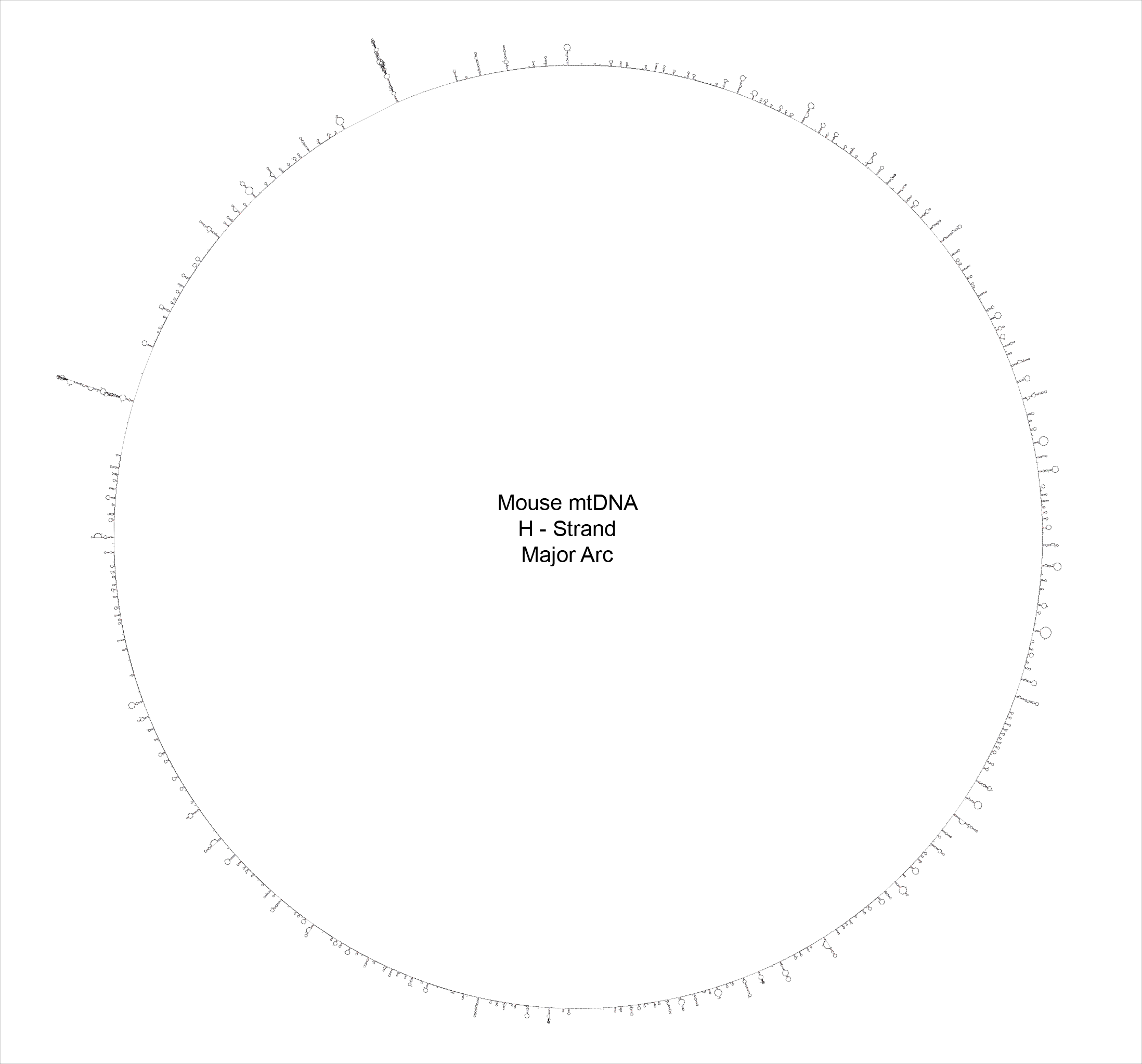

Supplement: Figure S8 — Stem-loop (SL) motifs in mouse mitochondrial genome. Abundance and distribution of predicted SL motifs in single-stranded mouse mtDNA heavy strand sequence from the end of D-loop till the beginning of L-strand origin of replication respectively. The minimum free energy folded structure is depicted in circular form. (TIF) [file pone.0035271.s008.tif]

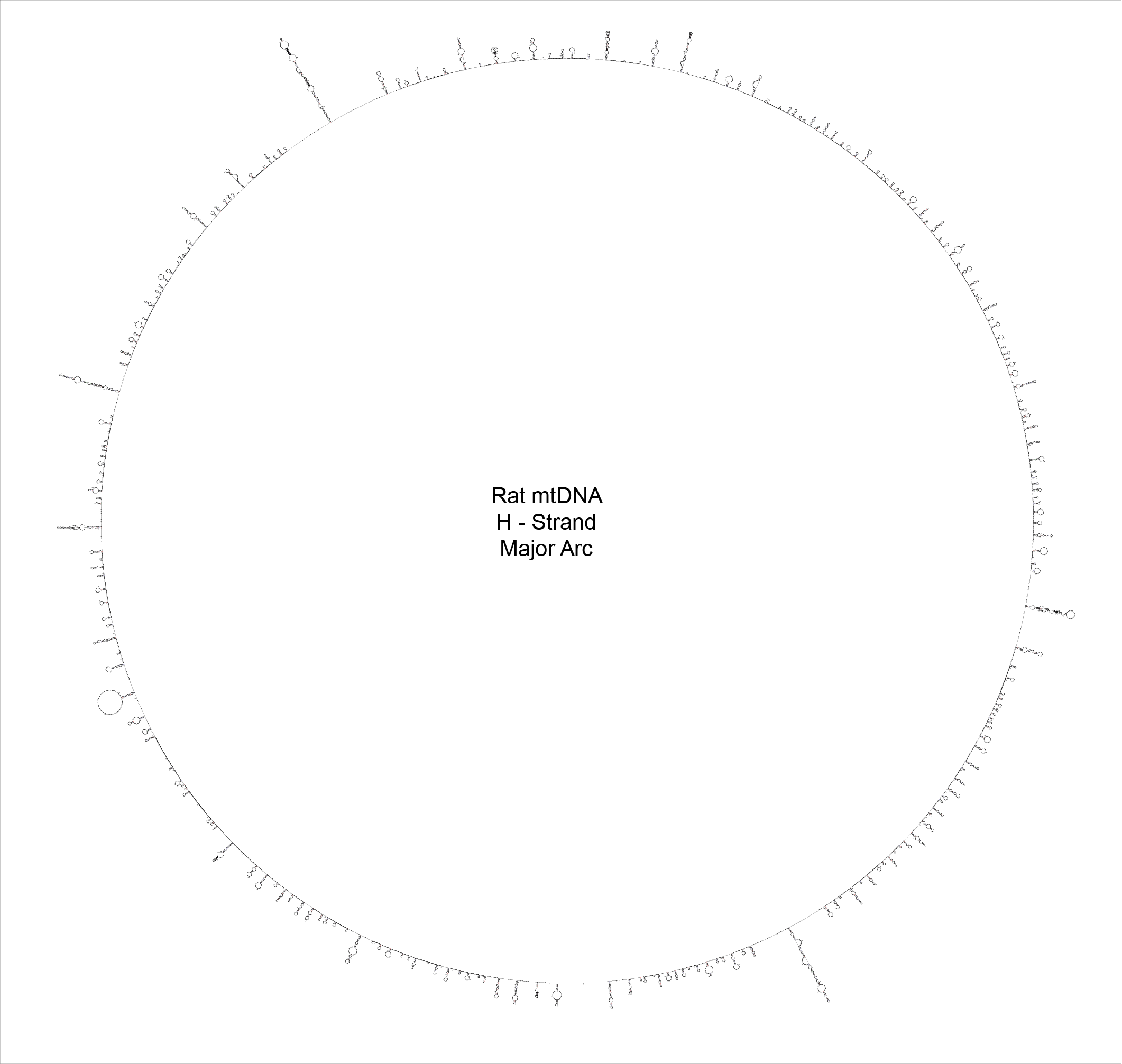

Supplement: Figure S9 — Stem-loop (SL) motifs in rat mitochondrial genome. Abundance and distribution of predicted SL motifs in single-stranded rat mtDNA heavy strand sequence from the end of D-loop till the beginning of L-strand origin of replication respectively. The minimum free energy folded structure is depicted in circular form. (TIF) [file pone.0035271.s009.tif]

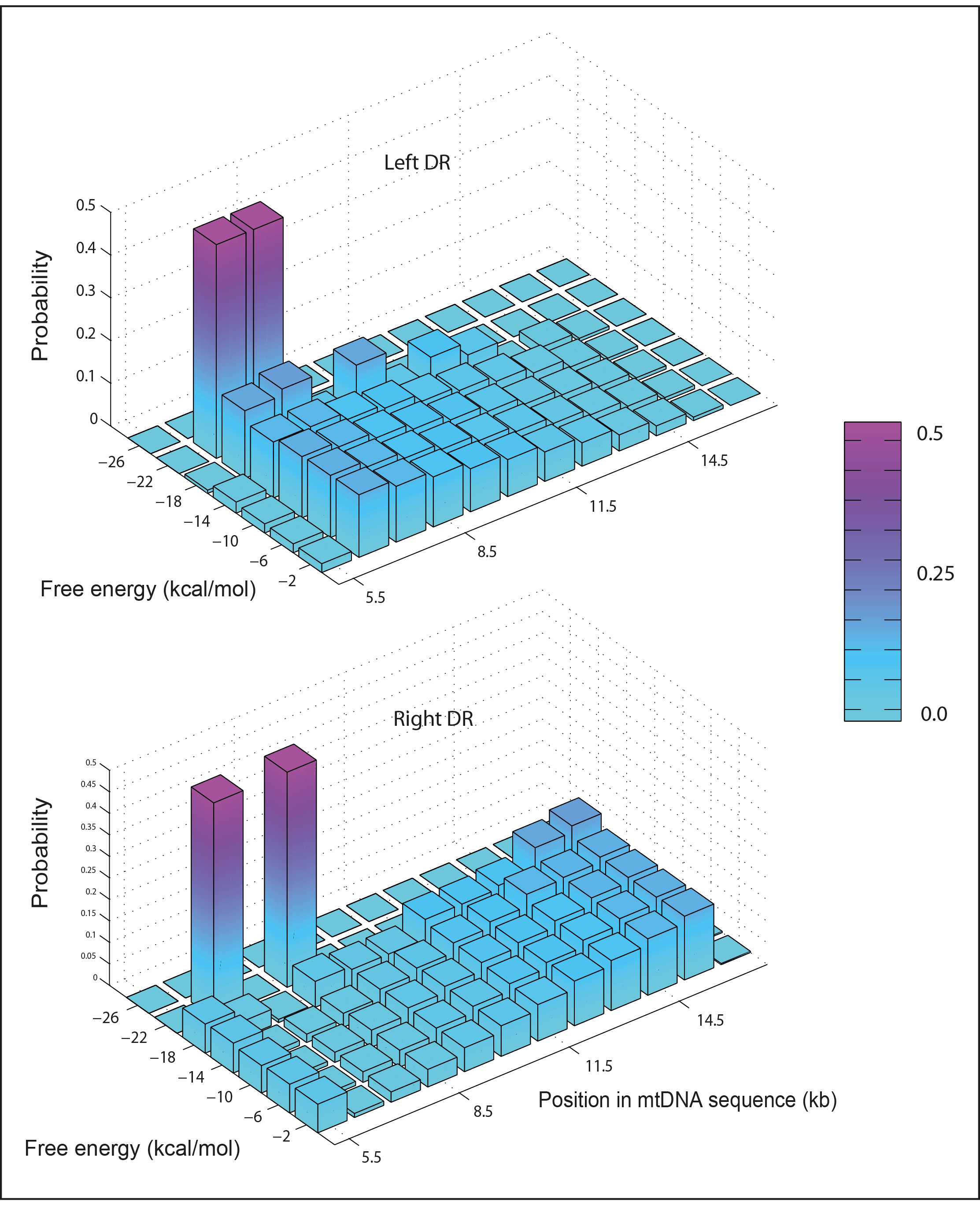

Supplement: Figure S10 — Free energy and position-wise distribution of the DRs. Resolution of DR distribution based on DR free energy and position in R1 random sequences (n = 100) of human mtDNA. The x- and y- axis values denote the midpoint of each corresponding bin, i.e. a bin centered at 5.5 kb denotes a range from 5 to 6 kb and similarly, a bin centered at −2 kcal/mol has a range between 0 to −4 kcal/mol. (TIF) [file pone.0035271.s010.tif]
