# Supplementary material for: Role of Direct Repeat and Stem-Loop Motifs in mtDNA Deletions: Cause or Coincidence?
Source: PLoS One. 2012 Apr 18;7(4):e35271. doi: 10.1371/journal.pone.0035271 (PMC3329436; doi:10.1371/journal.pone.0035271)
Supplement: Document S1 — Breakpoints data with reference. (PDF) [file pone.0035271.s011.pdf]

Supporting Document S1

Non Redundant Major Arc Deletions from Individual References

Human mtDNA Deletions

Number	Left Breakpoint	Right Breakpoint	Reference
1	8030	16071	[1]
1	8468	13580	[2]
1	7478	11439	[3]
2	11513	13809	
3	11738	15571	
4	6862	13060	
5	6340	14004	
6	9799	15556	
7	9920	16070	
8	6427	14100	
9	9665	15182	
10	8562	13953	
11	7181	14560	
12	9237	13053	
13	7722	14109	
14	7164	14109	
15	9758	13748	
16	10928	15524	

17	7398	13676	
18	9342	13145	
19	7395	12384	
20	9577	13952	
21	9243	14421	
22	8466	14362	
23	12083	15853	
24	7413	14570	
25	16157	16318	
26	7849	13473	
27	8554	13984	
28	6939	15450	
29	6521	15049	
30	10492	14751	
31	6329	13993	
32	10969	15535	
33	7856	12282	
34	11046	15850	
35	10931	15541	
36	7097	16071	
1	7682	13722	[4]
2	8469	13447	
3	7468	15989	

4	7817	16075	
5	7663	13804	
6	7398	13677	
7	7957	13370	
8	8131	13570	
9	7853	13887	
10	8299	13153	
11	8137	14771	
12	7912	9560	
13	8568	12976	
14	9570	10162	
15	10183	13756	
16	8525	13716	
17	7728	13806	
18	8030	14334	
19	8464	13138	
20	8441	14505	
1	9436	15913	[5]
2	8469	13447	
3	6173	16078	
1	8649	16084	[6]
1	8036	13095	[7]
2	6836	14380	

1	8637	16084	[8]
2	8470	13477	
1	6167	13052	[9]
2	6324	13989	
3	6501	13802	
4	6530	13831	
5	6919	13564	
6	7114	13992	
7	7125	13050	
8	7692	13983	
9	7808	12388	
10	8030	14563	
11	8231	13965	
12	8386	12820	
13	8469	13447	
14	8893	15503	
15	9487	13725	
16	9572	13938	
17	9737	14432	
18	9853	15019	
1	8482	13460	[10]
2	8421	13564	
3	8312	13667	

4	6601	16283	
1	7539	15237	[11]
2	7933	15604	
3	8256	15261	
4	8580	15239	
5	8580	15539	
6	8640	15369	
7	8663	15362	
8	8825	15658	
9	9535	15517	
10	9538	15658	
11	9538	15537	
12	6128	15434	
13	6468	15585	
14	8576	16075	
15	8601	15731	
16	8645	15656	
17	9621	15811	
18	9624	15434	
1	7805	13844	[12]
2	6555	14345	
3	6437	14077	
4	6427	15269	

5	8418	14127	
6	9924	15194	
7	6453	14288	
8	8408	14118	
9	8482	13460	
10	6423	14344	
11	6416	14182	
12	8049	14115	
13	6476	14146	
14	7815	13581	
15	7658	15548	
16	6585	15144	
17	6625	16074	
18	6759	15865	
19	8232	15542	
20	7821	13760	
21	7265	16035	
22	6790	15918	
23	7629	14813	
24	7808	14799	
25	8904	14903	
26	6835	14380	
1	8483	13459	[13]

2	8649	16084	
3	7960	13786	
4	8477	14811	
5	8440	16074	
6	7993	15729	
7	8035	16075	
8	7776	13532	
9	7842	13904	
10	8214	16069	
1	8470	13447	[14]
2	7954	13781	
3	8470	14805	
4	8367	16073	
5	8433	16068	
6	7986	15723	
7	8027	16071	
8	7769	13525	

Rhesus Monkey mtDNA Deletions

Number	Left Breakpoint	Right Breakpoint	Reference
1	6390	15657	[15]
2	6566	15750	
3	6517	15233	
4	6877	15588	
5	7474	15365	
6	8260	15141	
7	10209	15480	
8	10089	14826	
9	11025	15130	
10	6186	15806	
11	6114	15538	
12	6518	15242	
13	7534	15699	
14	6365	14489	
15	10133	15660	
16	8542	13064	
17	10172	14405	
18	5829	15710	
19	6534	15750	
20	8816	15202	
21	9064	14828	

1	7576	15603	[16]
2	7366	15381	
3	7363	15350	
4	7595	15564	
5	7453	15315	
6	7329	15184	
7	7449	15264	
8	7990	15391	
9	8114	15431	
10	9090	14834	
1	6116	10611	[17]
2	10596	16063	
3	9081	14828	

Mouse mtDNA Deletions

Number	Left Breakpoint	Right Breakpoint	Reference
1	8884	13120	[18]
2	9553	13279	
3	9089	12956	
1	9089	12956	[19]
2	8010	15061	
3	8001	15053	
4	7992	15045	
5	7702	14802	
6	7860	14988	
7	8497	14934	
8	7665	14812	
9	7708	15014	
10	7664	14606	
11	8166	14719	
12	7751	14614	
13	7759	14834	
14	8020	14900	
15	8039	14906	
16	7734	14649	
17	7669	14780	
18	7723	12757	

19	8118	14798	
20	8283	15065	
21	7731	15023	
22	7609	14669	
23	7695	12895	
24	7734	14658	
25	7736	12779	
26	7702	14568	
27	8295	14862	
28	7672	14604	
29	7691	14912	
30	7612	14656	
31	7740	15006	
1	7819	13641	[20]
1	7759	12454	[21]
1	8720	14116	[22]
1	7202	12531	[23]
1	9103	12976	
2	9058	12791	
3	9448	13103	[24]
4	8968	12890	
5	9374	13071	
6	8917	12784	

7	8932	12706	
8	9170	12648	
9	8904	12714	
10	8897	12292	
11	8982	12484	
12	8901	12890	
13	8956	12852	
14	9111	12516	
15	8878	12676	
16	8948	12719	
17	8953	12819	
18	8967	13056	
19	9020	12484	
20	9067	13086	
1	8883	13120	[25]
2	9554	13278	
3	9088	12956	
4	8992	13049	
5	8697	13301	
6	9262	13224	
7	8956	13334	
8	9627	13091	

Rat mtDNA Deletions

Number	Left Breakpoint	Right Breakpoint	Reference
1	7100	14392	[26]
2	6375	13084	
3	6883	14608	
4	10221	14991	
5	7166	14228	
6	9420	15112	
7	5530	14616	
8	6872	13867	
9	7268	14151	
10	6844	15225	
11	5935	14612	
12	7247	13095	
13	6336	13075	
14	6549	13914	
15	6033	14885	
16	5251	14984	
17	8149	14616	
18	6423	14545	
19	7398	15101	
20	5827	12360	
21	7170	14614	

22	6250	14399	
23	7810	15378	
24	7489	13122	
25	8201	14613	
26	6349	14219	
27	8313	15127	
28	7650	14466	
29	7501	11908	
30	6826	13410	
1	5173	15018	[27]
2	5188	15109	
3	5451	15109	
4	5531	14991	
5	6020	14911	
6	6061	14979	
7	6224	14911	
8	5250	14220	
9	5389	15265	
10	5457	14613	
11	5569	15080	
12	6879	16090	
13	8254	15129	
14	5242	14617	

15	6345	15299	
16	6099	14977	
17	5534	14404	
18	6248	14991	
19	6316	14908	
20	6099	14613	
21	7170	15136	
22	7454	15214	
23	5250	12893	
24	7169	14672	
25	7878	15292	
26	7525	14613	
27	6514	13190	
28	5658	13814	
29	7552	14933	
30	7686	14638	
31	7382	14215	
32	6749	13133	
33	8631	13943	
34	5423	15353	
35	6562	13621	
36	6099	13095	
37	6091	12806	

38	7837	13660	
39	7399	13212	
40	7689	12973	
41	9345	13801	
42	9394	13812	
43	10274	14612	
44	10307	14607	
45	5589	15120	
46	6117	13315	
47	6099	13178	
48	6099	13054	
49	6255	13315	
50	7493	13221	
51	7527	13100	
52	8098	14920	
53	8931	14920	
54	9662	13731	
55	10265	14219	
56	10427	14616	
57	10567	14610	
58	10896	13639	
59	11033	13707	
1	7599	15265	[28]

2	7654	15267	
3	7944	15265	
4	5377	15263	
5	5288	15266	
6	5459	15266	
7	7664	15142	
8	7653	14742	
9	7767	15422	
10	7768	14317	
11	8273	15224	
12	8104	15104	
13	8259	15296	
14	5458	15422	
15	5641	15289	
16	5379	15225	
17	7682	14590	
18	7734	15355	
19	5320	14983	
20	8465	15264	
21	8093	14395	
22	8204	15328	
23	7739	15265	
24	7959	15265	

25	7594	15398	
26	7652	15246	
27	7644	15265	
28	7847	15044	
1	7398	15101	[29]
2	7650	14466	
3	6026	14885	
4	6350	14218	

References

1. Baumer A, Zhang C, Linnane AW, Nagley P (1994) Age-related human mtDNA deletions: A heterogeneous set of deletions arising at a single pair of directly repeated sequences. Am J Hum Genet 54: 618-630.
2. Brierley EJ, Johnson MA, Lightowlers RN, James OF, Turnbull DM (1998) Role of mitochondrial DNA mutations in human aging: implications for the central nervous system and muscle. Ann Neurol 43: 217-223.
3. Bua E, Johnson J, Herbst A, Delong B, McKenzie D, et al. (2006) Mitochondrial DNA-deletion mutations accumulate intracellularly to detrimental levels in aged human skeletal muscle fibers. Am J Hum Genet 79: 469-480.
4. Eshaghian A, Vleugels RA, Canter JA, McDonald MA, Stasko T, et al. (2006) Mitochondrial DNA deletions serve as biomarkers of aging in the skin, but are typically absent in nonmelanoma skin cancers. J Invest Dermatol 126: 336-344.
5. Fayet G, Jansson M, Sternberg D, Moslemi AR, Blondy P, et al. (2002) Ageing muscle: clonal expansions of mitochondrial DNA point mutations and deletions cause focal impairment of mitochondrial function. Neuromuscul Disord 12: 484-493.
6. Hattori K, Tanaka M, Sugiyama S, Obayashi T, Ito T, et al. (1991) Age-dependent increase in deleted mitochondrial DNA in the human heart: possible contributory factor to presbycardia. Am Heart J 121: 1735-1742.
7. Khrapko K, Bodyak N, Thilly WG, Van Orsouw NJ, Zhang X, et al. (1999) Cell-by-cell scanning of whole mitochondrial genomes in aged human heart reveals a significant fraction of myocytes with clonally expanded deletions. Nucleic Acids Res 27: 2434-2441.
8. Kim UK, Kim HS, Oh BH, Lee MM, Kim SH, et al. (2000) Analysis of mitochondrial DNA deletions in four chambers of failing human heart: hemodynamic stress, age, and disease are important factors. Basic Res Cardiol 95: 163-171.
9. Kraytsberg Y, Kudryavtseva E, McKee AC, Geula C, Kowall NW, et al. (2006) Mitochondrial DNA deletions are abundant and cause functional impairment in aged human substantia nigra neurons. Nat Genet 38: 518-520.
10. Markaryan A, Nelson EG, Hinojosa R (2008) Detection of mitochondrial DNA deletions in the cochlea and its structural elements from archival human temporal bone tissue. Mutat Res 640: 38-45.
11. Nicholas A, Kraytsberg Y, Guo X, Khrapko K (2009) On the timing and the extent of clonal expansion of mtDNA deletions: evidence from single-molecule PCR. Exp Neurol 218: 316-319.
12. Reeve AK, Krishnan KJ, Elson JL, Morris CM, Bender A, et al. (2008) Nature of mitochondrial DNA deletions in substantia nigra neurons. Am J Hum Genet 82: 228-235.
13. Wei YH (1992) Mitochondrial DNA alterations as ageing-associated molecular events. Mutat Res 275: 145-155.
14. Zhang C, Baumer A, Maxwell RJ, Linnane AW, Nagley P (1992) Multiple mitochondrial DNA deletions in an elderly human individual. FEBS Lett 297: 34-38.
15. Gokey NG, Cao Z, Pak JW, Lee D, McKiernan SH, et al. (2004) Molecular analyses of mtDNA deletion mutations in microdissected skeletal muscle fibers from aged rhesus monkeys. Aging Cell 3: 319-326.
16. Lee CM, Eimon P, Weindruch R, Aiken JM (1994) Direct repeat sequences are not required at the breakpoints of age-associated mitochondrial DNA deletions in rhesus monkeys. Mech Ageing Dev 75: 69-79.
17. McKiernan SH, Colman R, Lopez M, Mark Beasley T, Weindruch R, et al. (2009) Longitudinal analysis of early stage sarcopenia in aging rhesus monkeys. Exp Gerontol 44: 170-176.
18. Brossas JY, Barreau E, Courtois Y, Treton J (1994) Multiple deletions in mitochondrial DNA are present in senescent mouse brain. Biochem Biophys Res Commun 202: 654-659.

19. Chung S, Eimon P, Weindruch R, Aiken J (1996) Analysis of age-associated mitochondrial DNA deletion breakpoint regions from mice suggests a novel model of deletion formation. AGE 19: 117-128.
20. Inoue K, Ito S, Takai D, Soejima A, Shisa H, et al. (1997) Isolation of mitochondrial DNA-less mouse cell lines and their application for trapping mouse synaptosomal mitochondrial DNA with deletion mutations. J Biol Chem 272: 15510-15515.
21. Inoue K, Nakada K, Ogura A, Isobe K, Goto Y, et al. (2000) Generation of mice with mitochondrial dysfunction by introducing mouse mtDNA carrying a deletion into zygotes. Nat Genet 26: 176-181.
22. Kim J, Kim M, Kwon IS, Song E (1997) Multiple age-associated mitochondrial DNA deletions in mouse brain. Journal of Biochemistry and Molecular Biology 30: 33-36.
23. Nelson I, Gerasimov S, Marsac C, Lestienne P, Boursot P (1993) Sequence analysis of a deleted mitochondrial DNA molecule in heteroplasmic mice. Mamm Genome 4: 680-683.
24. Vermulst M, Wanagat J, Kujoth GC, Bielas JH, Rabinovitch PS, et al. (2008) DNA deletions and clonal mutations drive premature aging in mitochondrial mutator mice. Nat Genet 40: 392-394.
25. Wang E, Wong A, Cortopassi G (1997) The rate of mitochondrial mutagenesis is faster in mice than humans. Mutation Research - Fundamental and Molecular Mechanisms of Mutagenesis 377: 157-166.
26. Cao Z, Wanagat J, McKiernan SH, Aiken JM (2001) Mitochondrial DNA deletion mutations are concomitant with ragged red regions of individual, aged muscle fibers: analysis by laser-capture microdissection. Nucleic Acids Res 29: 4502-4508.
27. Pak JW, Vang F, Johnson C, McKenzie D, Aiken JM (2005) MtDNA point mutations are associated with deletion mutations in aged rat. Exp Gerontol 40: 209-218.
28. Van Tuyle GC, Gudikote JP, Hurt VR, Miller BB, Moore CA (1996) Multiple, large deletions in rat mitochondrial DNA: evidence for a major hot spot. Mutat Res 349: 95-107.
29. Wanagat J, Cao Z, Pathare P, Aiken JM (2001) Mitochondrial DNA deletion mutations colocalize with segmental electron transport system abnormalities, muscle fiber atrophy, fiber splitting, and oxidative damage in sarcopenia. FASEB J 15: 322-332.
