# Supplementary material for: Role of Direct Repeat and Stem-Loop Motifs in mtDNA Deletions: Cause or Coincidence?
Source: PLoS One. 2012 Apr 18;7(4):e35271. doi: 10.1371/journal.pone.0035271 (PMC3329436; doi:10.1371/journal.pone.0035271)
Supplement: Document S2 — Average breakpoint-nearest DR distances in breakpoint data from individual studies. (PDF) [file pone.0035271.s012.pdf]

## Average breakpoint-nearest DR distances in breakpoint data from individual studies

| Reference | Tissue/ extraction | Number of deletions | Breakpoint-nearest DR distance distribution | | | p-value (One-sided z-test) |
|---|---|---|---|---|---|---|
| | | | Reported | Reshuffled ($n$ = 100) | | |
| | | | Mean | Mean | SD | |
| **Human** | | | | | | |
| Bua et al.,[1] | SM / SC | 36 | 10.3333 | 17.2285 | 1.4229 | $< 10^{-4}$ |
| Eshaghian et al., [2] | Skin / TH | 20 | 9.1750 | 17.0042 | 1.9633 | $< 10^{-4}$ |
| Kraytsberg et al., [3] | BR / SC | 18 | 7.1389 | 17.8486 | 2.0680 | $< 10^{-4}$ |
| Nicholas et al., [4] | SM / TH | 18 | 13.8333 | 17.3986 | 2.0723 | 0.0427 |
| Reeve et al., [5] | BR / SC | 26 | 11.5000 | 17.2783 | 1.7474 | 0.0005 |
| Overall | | 140 | 10.3929 | 17.3746 | 0.8016 | $< 10^{-4}$ |
| **Rhesus Monkey** | | | | | | |
| Gokey et al., [6] | SM / SC | 21 | 8.7619 | 17.7126 | 2.0371 | $< 10^{-4}$ |
| Overall | | 34 | 12.5147 | 17.3222 | 1.7778 | 0.0034 |
| **Mouse** | | | | | | |
| Chung et al., [7] | SM,BR/TH | 31 | 18.4032 | 18.1715 | 1.7953 | 0.5514 |
| Chung et al., [7] | SM / TH | 20 | 17.7500 | 17.2307 | 2.1634 | 0.5948 |
| Vermulst et al., [8] | SM / SC | 20 | 11.9000 | 16.4610 | 1.9429 | 0.0095 |
| Overall | | 62 | 15.5968 | 17.2474 | 1.1291 | 0.0719 |
| **Rat** | | | | | | |
| Cao et al., [9] | SM / SC | 30 | 17.9000 | 17.9857 | 1.8019 | 0.4810 |
| Pak et al., [10] | SM / SC | 20 | 15.2500 | 17.7110 | 2.2486 | 0.1369 |
| Pak et al., [10] | SM / TH | 26 | 16.0769 | 17.5465 | 1.7056 | 0.1944 |
| VanTuyle et al., [11] | BR / TH | 28 | 17.2679 | 18.6554 | 1.9702 | 0.2406 |
| Overall | | 118 | 16.5381 | 18.2033 | 0.9743 | 0.0437 |
| Overall (without Pak heart data) | | 104 | 16.7644 | 17.9636 | 0.9327 | 0.0993 |

**Note:** SM – Skeletal muscle, BR – Brain, SC – Single cell, TH – Tissue homogenate

**References**


1. Bua E, Johnson J, Herbst A, Delong B, McKenzie D, et al. (2006) Mitochondrial DNA-deletion mutations accumulate intracellularly to detrimental levels in aged human skeletal muscle fibers. Am J Hum Genet 79: 469-480.
2. Eshaghian A, Vleugels RA, Canter JA, McDonald MA, Stasko T, et al. (2006) Mitochondrial DNA deletions serve as biomarkers of aging in the skin, but are typically absent in nonmelanoma skin cancers. J Invest Dermatol 126: 336-344.
3. Kraytsberg Y, Kudryavtseva E, McKee AC, Geula C, Kowall NW, et al. (2006) Mitochondrial DNA deletions are abundant and cause functional impairment in aged human substantia nigra neurons. Nat Genet 38: 518-520.
4. Nicholas A, Kraytsberg Y, Guo X, Khrapko K (2009) On the timing and the extent of clonal expansion of mtDNA deletions: evidence from single-molecule PCR. Exp Neurol 218: 316-319.
5. Reeve AK, Krishnan KJ, Elson JL, Morris CM, Bender A, et al. (2008) Nature of mitochondrial DNA deletions in substantia nigra neurons. Am J Hum Genet 82: 228-235.
6. Gokey NG, Cao Z, Pak JW, Lee D, McKiernan SH, et al. (2004) Molecular analyses of mtDNA deletion mutations in microdissected skeletal muscle fibers from aged rhesus monkeys. Aging Cell 3: 319-326.
7. Chung S, Eimon P, Weindruch R, Aiken J (1996) Analysis of age-associated mitochondrial DNA deletion breakpoint regions from mice suggests a novel model of deletion formation. AGE 19: 117-128.
8. Vermulst M, Wanagat J, Kujoth GC, Bielas JH, Rabinovitch PS, et al. (2008) DNA deletions and clonal mutations drive premature aging in mitochondrial mutator mice. Nat Genet 40: 392-394.
9. Cao Z, Wanagat J, McKiernan SH, Aiken JM (2001) Mitochondrial DNA deletion mutations are concomitant with ragged red regions of individual, aged muscle fibers: analysis by laser-capture microdissection. Nucleic Acids Res 29: 4502-4508.
10. Pak JW, Vang F, Johnson C, McKenzie D, Aiken JM (2005) MtDNA point mutations are associated with deletion mutations in aged rat. Exp Gerontol 40: 209-218.
11. Van Tuyle GC, Gudikote JP, Hurt VR, Miller BB, Moore CA (1996) Multiple, large deletions in rat mitochondrial DNA: evidence for a major hot spot. Mutat Res 349: 95-107.
